# A Case of Myroides odoratimimus Bacteremia due to a Tunneled Dialysis Catheter-Associated Infection in a Paraplegic Patient

**DOI:** 10.1155/2023/3089837

**Published:** 2023-11-11

**Authors:** Carter Chapman, Barrett Meeks, Liam Ung, Jacob Nichols

**Affiliations:** Department of Internal Medicine, Infection Disease Division, Texas Tech University Health Sciences Center School of Medicine, 3601 4th St, Lubbock, TX 79430, USA

## Abstract

*Myroides odoratimimus* is a Gram-negative opportunistic pathogen known to rarely cause a wide range of opportunistic infections in humans. We report a novel case of *M. odoratimimus* bacteremia in a paraplegic patient with an extensive medical history likely due to a tunneled dialysis catheter infection that was successfully treated with levofloxacin.

## 1. Introduction

*Myroides odoratimimus* is a Gram-negative, nonmotile, nonfermenting, obligately aerobic bacillus characterized by yellow staining and fruity odor upon plating [1]. Previously categorized as *Flavobacterium odoratum*, this contaminated water and soil-dwelling organism does not present as part of the normal human flora and typically has little clinical significance; however, there are limited reports of infections in immunocompromised patients [2]. Patients with diabetes mellitus, chronic obstructive pulmonary disease, liver cirrhosis, and those receiving long-term corticosteroid therapy are at the highest risk. *Myroides odoratimimus* has thus been deemed an opportunistic pathogen, with the literature reporting urinary tract infections, soft tissue infections, and life-threatening bacteremia, most often in diabetic or immunocompromised patients [1–4]. However, there have been cases of infection in immunocompetent patients [2]. Further, extensive antibiotic resistance and biofilm formation make treatment and antibiotic therapy difficult [5]. We present a case of *M. odoratimimus* bacteremia due to a tunneled dialysis catheter infection in a paraplegic patient.

## 2. Case Description

A 65-year-old man with a past medical history of type 2 diabetes mellitus, hypertension, end-stage renal disease (ESRD) on hemodialysis Monday, Wednesday, Friday via an internal jugular tunneled dialysis catheter, and a spinal cord injury in 1969 resulting in paraplegia and neurogenic bladder was admitted in October of 2022 for weakness and generalized pain after missing a dialysis session since discharge from a recent hospitalization. His CKD was diagnosed in 2008 and was determined to be secondary to obstructive uropathy. In July of 2021, he underwent hemodialysis (HD) induction via placement of a tunneled right internal jugular catheter. This tunneled dialysis catheter was manipulated and exchanged once prior to his admission in October of 2022. In March of 2022, the patient engaged in a discussion regarding creation of an arteriovenous fistula (AVF) or an arteriovenous graft (AVG) for long-term dialysis needs; however, this fistula was never created for unclear reasons. During the time leading up to his admission, the patient had a known history of nonadherence to his HD schedule. There is no documentation of a discussion of peritoneal dialysis, however given the patient's social situation–living alone, a history of medical self-neglect, and consistent refusal to accept medical assistance–it is not likely that he would have been an appropriate candidate for this form renal replacement therapy (RRT). There is no known or documented history of vascular access infections.

On admission, he was found to have multiple unstageable wounds over his torso, bilateral upper and lower extremities, and sacrum. Labs drawn demonstrated significant leukocytosis as well as electrolyte derangements (hyponatremia, hyperkalemia, and hypocalcemia) and uremia consistent with uncontrolled ESRD. Blood and urine cultures were obtained. In the emergency department (ED), the patient was given a one-time dose of vancomycin and piperacillin/tazobactam then started on cefazolin 1 g IV q8. He was then started on vancomycin with ESRD dosing protocol. Blood cultures drawn on admission were shown to be positive for Gram-negative rods, with sepsis polymerase chain reaction (PCR) resulting positive for CTX-B *K. pneumoniae*. Given this result, he was started on meropenem 1 g IV q24. Imaging demonstrated possible osteomyelitis of the coccyx. The day following admission, cultures were obtained from his sacral wound. Urine cultures obtained during admission grew at least three organisms, while blood cultures officially grew *Klebsiella pneumoniae* two days after admission. Given his bacteremia, it was determined that his internal jugular tunneled dialysis catheter needed to be removed. An initial attempt by interventional radiology was unsuccessful, necessitating removal by vascular surgery. Prior to removal, the tip of the tunneled dialysis catheter was used to obtain a set of repeat blood cultures.

Three days after admission, blood cultures were found to grow *Myroides odoratimimus*. At this time, minocycline was started for the treatment of *M. odoratimimus*. The following day, susceptibilities for *M. odoratimimus* returned and showed sensitivity to levofloxacin, so minocycline was discontinued, and levofloxacin was started. Given that the CTX-B *K. pneumoniae* that grew on admission was also found to be sensitive to levofloxacin and that the patient was likely not benefiting from vancomycin based on blood culture results, meropenem and vancomycin were discontinued the following day, and levofloxacin was kept as therapy for both CTX-B* K. pneumoniae* and *M. odoratimimus*. Initial cultures from his sacral wounds grew only normal skin flora; however, repeat cultures of the patient's sacral wound were obtained and grew MRSA, for which clindamycin was initially started. However, given the gross appearance of the wound, this isolate was concluded to likely be normal flora and nonpathologic, so clindamycin was discontinued after one day of treatment. Repeat blood cultures were negative, and levofloxacin was discontinued after 5 days of therapy. Given negative repeat blood cultures after removal of his tunneled dialysis catheter, this was likely the source of his bacteremia. The contamination pathway of the catheter with *M. odoratimimus* is not clear. However, given the fact that *M. odoratimimus* resides in contaminated water and in environmental soil, it is presumed that contact with one of these sources resulted in infection. Given the patient's history of medical self-neglect, poor personal and medical hygiene, and the state and appearance of his skin on admission, it is likely that these factors contributed to contamination of his catheter.

After removal of the patient's tunneled dialysis catheter, he refused placement of a new catheter and stated that he no longer wanted to receive dialysis. After lengthy discussions with the patient and his family and involvement of Palliative Care, he decided to transition to hospice care and was discharged to an outpatient hospice facility.

## 3. Discussion

*M. odoratimimus* exhibits extensive antibiotic resistance, through both not yet known and recently elucidated mechanisms. It has been shown that *M. odoratimimus*, as well as other *Myroides* species, produce a wide-spectrum B-lactamase that, in most cases, renders the overwhelming majority of B-lactam antibiotics ineffective. In addition, there have been cases of *M. odoratimimus* exhibiting resistance to aminoglycoside and fluoroquinolone antibiotics, as well as sulfamethoxazole [6]. Further complicating treatment, especially in wound infections, is the ability of *M. odoratimimus* to form biofilms [6].

In our case, empiric treatment for *M. odoratimimus* was initially with minocycline, as previously reported cases demonstrate that *M. odoratimimus* isolates are unanimously sensitive to this antibiotic [7]. However, once susceptibility testing resulted, the isolate in our case was found to be sensitive to levofloxacin with MDIL ≤ 0.5 and trimethoprim-sulfamethoxazole with MDIL 2/38. Given this result, and the previous susceptibility testing of CTX-B *K. pneumoniae* isolated from the patient's blood cultures also showing sensitivity to levofloxacin with an MDIL of ≤0.5, levofloxacin was chosen as antibiotic coverage for both organisms.

An extensive literature review reveals that *M. odoratimimus* infections in mostly immunocompromised patients, although there is at least one case that occurred in an immunocompetent child after a pig bite [2]. These infections include, but are not limited to, an outbreak of urinary tract infections, a urinary tract infection secondary to prolonged catheterization, and a case of bacteremia secondary to a chronic diabetic foot wound [2, 4, 6]. To our knowledge, this is the first case of *M. odoratimimus* bacteremia secondary to a tunneled dialysis catheter.

This case adds to the limited, but growing body of literature of *M. odoratimimus* infection by presenting a novel patient population and source of infection. In addition, given that antibiotic treatment of *M. odoratimimus* can be quite difficult due to the extensive antibiotic resistance detailed above and that the incidence of *M. odoratimimus* appears to be increasing, our case contributes to the literature because the isolate described in our case was sensitive to levofloxacin, which was the first report case of such a susceptibility. Lastly, our case highlights the importance of proper hygiene and management of tunneled dialysis catheters, as they can serve as sources of infection [8].

Both in the United States and globally, the incidence and prevalence of renal failure and the resultant need for long-term RRT continue to increase annually. Tunneled dialysis catheters, which are a form of central venous catheter (CVC), are one of the options for vascular access in patients receiving hemodialysis–the most common form of RRT–with other options including AV fistula formation and AV graft placement. Tunneled dialysis catheter use for HD is associated with high rates of morbidity and mortality due to its various complications, including infection with resulting bacteremia and venous stenosis with resulting lack of patency. Because of these complications, CVC use for HD is associated with higher rates of cardiovascular events, all-cause mortality, and fatal infection than both AV fistula and AV graft use [9]. In a prospective cohort study assessing the rate of complications, including bacteremia, other infections, central venous stenosis or thrombosis, and other CVC-related complications, it was found that 30% of patients studied experienced a complication at one year and 38% of patients studied experienced a complication at two years. In this study, bacteremia was found to be responsible for 72% of CVC-related hospitalizations and occurred in 10% of the patients studied, indicating that bacteremia was the greatest source of morbidity for patients using a CVC for HD [10]. Despite increased morbidity and mortality and recognized clinical inferiority to AV fistula and AV graft use, the prevalence of CVC use still varies significantly based on geography; in Europe, Japan, Australia, and New Zealand, there is a much lower prevalence of CVC use and a much higher prevalence of fistula use, while in North America, CVC use is much greater than fistula use [9].

## 4. Conclusion

*Myroides odoratimimus* is a Gram-negative opportunistic pathogen known to cause urinary tract infections, skin and soft tissue infections, and life-threatening bacteremia, primarily in immunocompromised patients. Due to its genetically encoded extensive antibiotic resistance and its ability to form biofilms, it is often difficult to treat appropriately. This case report describes a case of *M. odoratimimus* bacteremia in a paraplegic patient secondary to a tunneled internal jugular dialysis catheter-associated infection that was successfully treated with levofloxacin, which was the first reported case to our knowledge detailing levofloxacin susceptibility and therapy. This case also serves to add to the extensive body of literature concerning the complications of tunneled dialysis catheters and the clinical decision algorithm used to decide which form of vascular access is most appropriate for patients with CKD requiring renal replacement therapy.

## Data Availability

All data used to support the findings of this study are included within the article.
